# A pilot study to evaluate the feasibility and acceptability of a mobile phone counselling intervention for perinatal women living with HIV and depression in India

**DOI:** 10.1093/oodh/oqaf012

**Published:** 2025-05-21

**Authors:** Mona Duggal, Avina Sarna, Neha Dahiya, Roopal Jyoti Singh, Sangchoon Jeon, Anuradha Subramanian, Mamta Sood, Pushpendra Singh, Gurjinder Kaur, Nancy R Reynolds

**Affiliations:** National Institute for Research in Digital Health and Data Science, Indian Council of Medical Research (ICMR), V. Ramalingaswami Bhawan, P.O. Box No. 4911 Ansari Nagar, New Delhi, 110029, India; Population Council, Zone 5A, Ground Floor, India Habitat Centre, Lodi Road, New Delhi, 110003, India; Division of Implementation Research, Indian Council of Medical Research (ICMT), V. Ramalingaswami Bhawan, P.O. Box No. 4911 Ansari Nagar, New Delhi, 110029, India; Population Council, Zone 5A, Ground Floor, India Habitat Centre, Lodi Road, New Delhi, 110003, India; Yale School of Nursing, 400 West Campus Drive, Orange, CT, 06477, USA; Department of Medicine, Maulana Azad Medical College, 2 Bahadur Shah Zafar Marg, New Delhi, 110002, India; Department of Psychiatry, All India Institute of Medical Sciences, 4th Floor, Teaching Block, Ansari Nagar, New Delhi, 110029, India; Indraprastha Institute of Information Technology, Delhi Okhla Industrial Estate, Phase III, New Delhi, 110020, India; Department of Hematology, Postgraduate Institute of Medical Education and Research, Room No. H-1, Research Block A, Madhya Marg, Sector 12, Chandigarh, 160012, India; Johns Hopkins University School of Nursing, 525 N. Wolfe Street, Baltimore, MD, 21205, USA

**Keywords:** depression, HIV, mobile phone counselling, women, perinatal, pregnant

## Abstract

Pregnant and postpartum women living with Human Immunodeficiency Virus (HIV) are at high risk for perinatal depression, which can significantly undermine HIV care engagement and treatment outcomes. Despite this, depression often remains unidentified and untreated. This pilot study evaluated the feasibility, fidelity, acceptability and preliminary effects of a mobile phone counselling intervention among perinatal women living with HIV and depression in India. Forty women in their third trimester (≥28 weeks of gestation) and screening positive for depressive symptoms (Edinburgh Postnatal Depression Scale ≥13) were recruited from three government-run treatment centres in Delhi. Participants were randomized to: (a) the intervention condition, ‘BEST-ma-CARE’ mobile phone counselling, or (b) an attention control condition, time- and phone-matched perinatal wellness counselling. All counselling was delivered via basic mobile phones. Data were collected at baseline and follow-up at 36–40 weeks pregnancy, within 7 days of delivery and at 6 and 12 weeks postpartum. Overall, 82% of the participants completed the study, with higher retention in the intervention arm (90%). Engagement was also higher in the intervention group with 78% of antenatal and 95% of postnatal calls attended, compared to 70% and 80% in the control group. The intervention was highly acceptable; 76% reported it ‘helped them a lot’, compared to 69% in the control group. All participants (100%) appreciated scheduled counsellor calls. Both groups showed reductions in depressive symptoms and perceived illness severity, while internalized stigma increased only in the control group. Improvements in antiretroviral adherence, viral load and CD4 counts were stronger in the intervention group. Results indicate that integration of mobile phone counselling into maternal HIV care shows promise.

## INTRODUCTION

Globally, over 50% of the 39.9 million people living with Human Immunodeficiency Virus (HIV) are women in low- and middle-income countries (LMICs), and most are of childbearing age [1, 2]. Although perinatal transmission still results in a significant number of infants infected with HIV worldwide, antiretroviral therapy (ART) use among pregnant women infected with HIV can significantly reduce transmission rates to <2% [3].

However, women living with HIV experience significantly higher levels of antenatal and postnatal depressive symptoms compared to their HIV-negative counterparts [4]. When present, depression in this population can severely compromise both maternal and child health outcomes [5, 6]. Depression is a robust predictor of poor care seeking and retention in care, non-adherence to HIV medications, adverse clinical outcomes and HIV disease progression [7–10]. Of particular concern are the unmet mental health care needs of perinatal women with comorbid depression and HIV in low-resource settings. This has received little attention, and there is limited scientific evidence available to guide provision of care. Identifying methods to effectively improve women’s health behaviours and linkages to care without heightening their burden is a critical clinical challenge [6, 11–13].

India provides an opportune setting to study the potential of a practical, integrated and contextualized approach. More than 22 000 pregnant women are infected with HIV in India [14, 15]. The levels of prenatal depression reported in the Indian subcontinent range from 25% to 45%, with pilot data showing rates as high as 40–84% among perinatal women with HIV [16, 17]. Women in India often face gender-related sociocultural challenges and obstacles to attaining and maintaining appropriate care for themselves and later their infants. Limiting factors include stigma, low education and knowledge about treatment options, low mobility and access to care [4, 18–24]. There is a need for practical, scalable strategies that integrate mental health support into existing prevention of mother-to-child transmission (PMTCT) programs [4, 25–30]. This study examined the feasibility and acceptability of a mobile phone counselling intervention, BEST-ma-CARE [Better Education, Support, Treatment for maternal Capacity, Adherence, Retention in Care], for perinatal women living with HIV and depression in Delhi, India. The BEST-ma-CARE intervention was developed for this study and is guided by self-regulation theory, focusing on patient-centered counselling to promote coping and self-care behaviors.

## MATERIALS AND METHODS

### Study design

**Figure 1:**
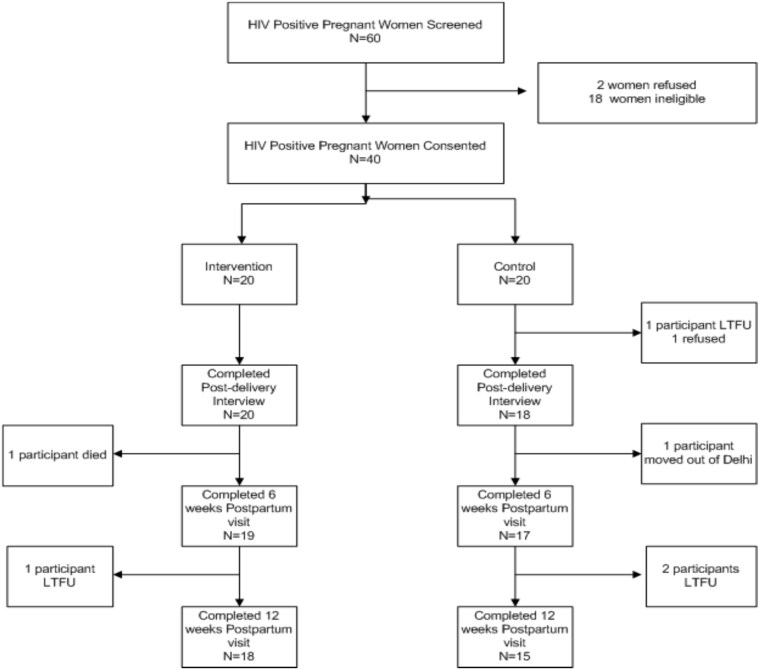
Participant retention.

This Stage Ib pilot trial [31] employed a two-arm randomized between-group design to examine the feasibility, fidelity, acceptability and preliminary effects of the BEST-ma-CARE intervention in comparison to a time-matched control to test trial procedures and processes to prepare for a future full-scale efficacy trial. Participants were randomly assigned to either the treatment group (BEST-ma-CARE mobile phone counselling intervention) or an attention-matched control group (perinatal wellness counselling). The study was conducted among perinatal women living with HIV and depression, receiving PMTCT care from one of three government antiretroviral treatment centres (ARTCs) in Delhi, India. In both study arms, low-cost mobile phones were provided as a component of the study for delivery of one-to-one counselling with the study counsellor over time. Data collection occurred at baseline and at four follow-up time points over 12 weeks post-randomization (Table 1, Fig. 1). Our primary thesis was that the BEST-ma-CARE intervention would be feasible and acceptable for women living with HIV infection and depression in India. In addition, we expected the fidelity of the intervention to be maintained, and, in keeping with the guiding conceptual framework, there would be a positive relationship between the BEST-ma-CARE intervention and women’s psychosocial and biologic outcomes (depression, medication adherence, illness perceptions, stigma, HIV-1 RNA and CD4).

**Table 1:** Summary data collection

**Variables**	**Measure/Source**	**Data collector**	**Baseline/Pre-randomization**	**Follow-up/Post-randomization**			
			**~ 28-wks of gestation**	**36–40-wks gestation**	**Within 1-wk delivery**	**6-wks postpartum**	**12-wks postpartum**
Feasibility	Study-specific forms/Research assistant	Research Assist	x	x	x	x	x
Fidelity	Study-specific call logs/Counsellor	Research Assist		x	x	x	
Acceptability	Study-specific forms/Participant	Research Assist					x
Socio-demographic characteristics	Study-specific forms/Participant	Research Assist	x				
Psychosocial indicators							
Depressive symptoms	EPDS/Participant	Research Assist	x	x	x	x	x
Antiretroviral Adherence	Adherence self-report/Participant treatment interruption/Participant; MPR/Pharmacy refill	Research Assist	x			x	x
Illness perceptions	B-IPQ/Participant	Research Assist	x			x	x
Stigma	HHS/Participant	Research Assist	x			x	x
Biologic							
Virologic	HIV-1 RNA/Participant	SRL labs	x			x	
Immunologic	CD4 count/Participant	SRL labs	x			x	

### Study setting

The study was conducted at the ARTCs at three government hospitals in Delhi, India (Lok Nayak Jaiprakash, Ambedkar and Lal Bahadur Shastri hospitals). All three ARTCs provided similar PMTCT services with regard to the provision of antiretroviral treatment (ART) and client follow-up in accordance with national guidelines. A typical ARTC is run under the mandates of the National AIDS Control Organisation (NACO). The human resources at each ARTC included a Senior Medical Officer (SMO), Medical Officer (MO), lab technician, counsellors, pharmacists, data managers, staff nurses, institutional nurses and care coordinators.

Hospitals in Delhi provide healthcare to Delhiites and people from the neighbouring states of Haryana, Uttar Pradesh and Rajasthan. Thus, Delhi offered a wide variety of potential participants with diverse cultural and social differences, perceptions about treatment for HIV, depression and difficulties faced in continuing treatment. These factors are significant and necessary for establishing the feasibility of an integrated mobile-based counselling intervention to facilitate linkage and adherence to HIV and depression treatment.

### Sample size

The sample size was set at 40 participants (20 participants per arm) based on the guidelines for Stage 1b pilot studies, which suggest that 15–75 participants per arm are adequate for assessing feasibility and acceptability, testing trial procedures, predicting likely dropout rates and preliminary trend in effect. However, these studies are not powered to assess the superiority of the intervention [31–33].

### Eligibility criteria

The study included pregnant women living with HIV in their third trimester of pregnancy (≥28 weeks delivery, ≥18 years of age), who were receiving ART treatment or prophylaxis, who screened positive for depressive symptoms (≥13 on the Edinburgh Postnatal Depression Scale (EPDS) [34]), were able to communicate in English or Hindi and were able to provide informed consent. EPDS scores range from to 0 to 30. A score of 13 or higher indicates that the mother is likely to suffer from a depressive illness [34, 35]. Exclusion criteria included: (i) suicidal ideation as detected at time of screening, (ii) psychotic symptoms detected at time of screening, (iii) unable or unwilling to remain in the local area for the following 24 weeks and (iv) any other condition that, in the opinion of the study interviewer or ARTC site provider, would compromise the candidate’s ability to participate in the study.

### Recruitment, enrollment, randomization and blinding procedures

Candidates were invited to consider participation by a trained site staff member, where the study was fully explained, and upon verbal consent, a screener questionnaire was administered, which included the EPDS tool [34] to assess depression. Written consent for study participation was obtained from eligible candidates. Participation in the study was voluntary; participants were informed that they could withdraw their participation at any time without penalty. After screening and obtaining written consent, baseline measures were collected, which included a contact form, participant characteristics and psychosocial and biologic measures. Upon completion of the baseline measures, the participants were randomly assigned to the intervention or control arm. Randomization was performed using a random number generator in Microsoft Excel, and a corresponding unique patient ID (PID) was remotely generated for each participant by a statistician. Research assistants who collected the data were blinded to the assigned arm of the participants. Study counsellors could not be fully blinded to the intervention assignment but were not informed which intervention was the treatment and which was the control condition.

### Study interventions

Both study arms received usual care through the ARTCs, where participants were enrolled and data were collected. Usual care included ART medication adherence counselling but not routine depression screening or mental health counselling. In both arms, the call schedule comprised two calls in the first week after recruitment and one call every 2 weeks until delivery (a total of seven to eight scheduled calls in the antepartum period), and one call in the first week after delivery, followed by one call every 2 weeks (total of six scheduled calls in the postpartum period). The counsellor made up to six attempts for each scheduled contact. Participants also had the option to initiate calls between scheduled calls to confer with the study counsellor on health-related matters.

Trained study counsellors (one counsellor per study arm) to deliver counselling to the study participants. The study counsellors were non-specialists with backgrounds in sociology and social work (MSW) and past work experience in counselling vulnerable populations. The study counsellor for the intervention arm was trained in the intervention delivery and nuances of depression in the peripartum period, especially among women living with HIV, by a psychiatrist from the All India Institute of Medical Sciences (AIIMS), Delhi.

### Intervention group

Participants in the intervention arm received the BEST-ma-CARE intervention, a theory-guided, empirically supported and manualized counselling program adapted for women living with HIV in India [22, 36–40].

#### Theoretic framework

The intervention is grounded in Self-Regulation Theory [36], as applied to HIV care [22, 37–41]. At its core, the intervention employs a structured, patient-centred counselling approach to build participants’ capacity to initiate and sustain effective coping strategies and self-care behaviours. Effective self-care is essential to managing chronic conditions like HIV, where much of the daily care occurs outside formal healthcare settings. Individuals must routinely make health-related decisions that are influenced by how they understand and interpret their illness—referred to as *illness representations* [36]. These representations are shaped by personal, cultural and contextual factors that often diverge from biomedical models. They influence how individuals cope with illness, adhere to treatment and ultimately affect health outcomes. Illness representations are not always aligned with biomedical models and can significantly shape coping strategies, treatment adherence and ultimately, health outcomes. As individuals encounter new health information, it is filtered through these internal schemas—accepted or rejected depending on alignment with existing beliefs.

BEST-ma-CARE therefore emphasizes personalized assessment of each participant’s illness representations and life circumstances. Counsellors use this insight to identify areas of risk—such as stigma, side effects, depressive symptoms or competing life priorities—and deliver health information and emotional support that are tailored to the individual’s cognitive, emotional and situational context. This tailored approach makes the intervention content more relevant, meaningful and actionable—facilitating problem-solving and emotional regulation in the face of complex health challenges. Importantly, self-care is conceptualized not as a one-time action but as a dynamic, ongoing process requiring adaptation and problem-solving as new challenges arise. Depression, for instance, is a significant and common barrier to consistent self-care that may ebb and flow among individuals living with HIV over time.

#### Mechanisms of action

Within this framework, the intervention promotes self-care through three key mechanisms: (i) delivery of timely, personalized health information aligned with the individual’s illness representation and evolving needs; (ii) development of behavioral and emotional coping skills to address barriers and support for effective health-related decision-making; and (iii) establishment of a supportive therapeutic relationship with a trained provider. Together, these components foster both intrapersonal and interpersonal efficacy and enhance the individual’s ability to adaptively manage their illness within the constraints of their environment and relationships.

#### BEST-ma-CARE delivery

The counselling sessions were delivered proactively by the study counsellor. Sessions were interactive, guided by theory-based, open-ended prompts and customized to each participant. Key components included assessing the participant’s understanding of HIV and ART, identifying risks to adherence and well-being and collaborative problem-solving to enhance engagement in care. Counsellors aimed to shift maladaptive illness perceptions, present new knowledge and skills and support the development of alternative, health-promoting behaviours using empathetic, non-judgmental communication. Sessions were conducted over time, allowing reinforcement and coaching through evolving challenges. The frequency, duration and specific content of calls were individualized based on participant needs and tapered over time. Additional details on the intervention are available upon request (nancy.reynolds@jhu.edu).

### Control group

Participants in the control group received proactive calls from the counsellor, which were time- and attention-equivalent to the experimental condition. This was performed to control for the attention effect of providing mobile phones and regular calls by a counsellor in the intervention arm. In the control arm, calls from the counsellors focused on infant and maternal nutrition-related health education per national guidelines; no theory-guided HIV- or PMTCT-related counselling was provided as a component of the calls.

### Measurement

We used protocol-specific tools to measure ‘feasibility, fidelity and acceptability’ of the intervention and study protocol. We used protocol specific tools to measure participant ‘socio-demographic characteristics’ and standardized instruments—that have been previously translated and tested in India or resource-poor settings and are congruent with the guiding theoretical concepts—to measure ‘psychosocial and biologic’ outcomes (‘depression, medication adherence, illness perception stigma, viral load and CD4’) (see summary in Table 1).

#### Feasibility, fidelity and acceptability measures

‘Feasibility’ was measured by: (i) the ratio of eligible study participants to those enrolled; (ii) the number of scheduled study visits completed; (iii) attrition between baseline and follow-up; and (iv) reasons for premature drop-out. ‘Fidelity’ was assessed through: (v) the number of scheduled phone calls completed on time; (vi) participation in intervention sessions, including the total number of sessions, number of sessions completed without break offs, length (minutes) of sessions; and (vii) congruence of between call content and the intended protocol (Table 1).

Data for items 1–4 were collected by a research assistant during routine clinic visits at baseline and follow-up. Data for items 5–7 were obtained from call logs completed by study counsellors after each counselling call in both arms. These logs included the date, duration and content of each session. Counsellors also recorded brief qualitative notes summarizing participant responses and key discussion points.

‘Acceptability’ was evaluated using a structured tool administered at endline (12 weeks postpartum) by a research assistant. This tool captured participant satisfaction with: (i) overall participation in the study; (ii) the quality, duration and frequency of counselling calls; (iii) relevance of topics covered; (iv) the content, mode of delivery and structure of the intervention; (v) perceived personal benefits; and (vi) anticipated benefits for other women with HIV.

#### Baseline and follow-up psychosocial and biologic outcome measures (preliminary trend in effect)

Baseline data (collected in participants’ third trimester) included information on socio-demographic characteristics and psychosocial (depression, adherence, illness perceptions and stigma) and biologic (HIV-1 RNA and CD4 counts) indicators. Follow-up data were collected post-randomization at 36–40-weeks pregnancy, within 7 days of delivery and at 6 and 12 weeks postpartum (see specifics in Table 1).

‘Depression’ was measured using the Edinburgh Postnatal Depression Scale [34], a 10-item tool validated in the Indian sociocultural context. Scores of 13 or higher are considered indicative of depression or a high probability of experiencing depression. The measure exhibits good internal consistency, with a Cronbach’s alpha coefficient typically ranging from 0.77 to 0.86 [35].

‘Adherence’ was assessed in three ways: (i) Self-reported adherence was assessed using the ACTG Adherence Questionnaire [42]. The validated and widely used questionnaire queries the participant on the number of doses missed by a medication during each of the 4 days before a clinic visit (e.g. ‘How many doses did you miss yesterday, the day before yesterday, 3 days ago and 4 days ago?’); (ii) self-reported treatment interruptions (TIs), defined as discontinuation of ART for ≥7 days without medical indication; and (iii) medication possession ratio (MPR). The MPR was derived from pharmacy refill information collected from pharmacy registers and recorded as a percentage: MPR = number of days participants had a supply of medications/number of days in the study.

‘Illness perceptions’ were assessed using the nine-item validated Brief Illness Perception Questionnaire (B-IPQ) [43]. The tool is designed to rapidly assess the cognitive and emotional representations of illness with scores ranging from 0 to 80; higher scores indicate more health concerns and poorer perception of health. Studies have shown internal consistency coefficients (Cronbach’s alpha) ranging from 0.74 to 0.94 [43].

‘Stigma’ was assessed using the 16-item HIV Stigma Scale (HSS) [44, 45]. It is composed of items that assess different aspects of stigma, including internalized stigma, concerns about public attitudes, negative self-image and disclosure concerns —each represented by four items. The scale is scored on a 4-point Likert scale [1–4], yielding a possible score range from 16 to 64, with higher scores indicating greater perceived stigma. Internal consistency coefficients (Cronbach’s alpha) for the 16-item version of the HSS have been reported as 0.88 [45].

‘Viral load/CD4 labs’ were collected by SRL Diagnostics® at baseline and 6 weeks postpartum (collection details below).

### Data collection procedures and management

Participant data were collected face-to-face during routine clinical visits by research assistants. Each interview took ~40 minutes to 1 hour. A minimal reimbursement of Rs. 250 (~$3.35 USD) was provided for each face-to-face interview. A data collection schedule was included in the individual patient study files. Members of the research team monitored the data quality throughout the study with regular and frequent reviews of monthly reports to identify inconsistencies, extreme values or invalid codes.

Participant data and counsellor call log data were collected on paper forms and entered into a tablet laptop by a research assistant using unique identifiers. Personal information needed for tracking and informed consent was stored separately from the other data in a locked cabinet.

The viral load (HIV-1 RNA) and CD4 cell counts were collected by SRL Diagnostics®, which have an extensive network of labs across Delhi. At baseline, after screening was completed and a participant was found eligible and enrolled, the research assistant contacted a phlebotomist from SRL laboratories nearest to the study site. While the baseline assessment was being administered to the participants, the phlebotomist arrived at the study location and collected a blood sample after obtaining written informed consent. This process was repeated at 6 weeks postpartum. A blood report was published by SRL Diagnostics® for each participant and shared with the study team electronically, within 3–4 days of blood collection.

### Statistical analyses

Preliminary analyses included summarizing the appropriate distributional properties of all baseline characteristics for the groups, including sample mean, standard deviation, median and frequency. Descriptive analyses were applied to summarize survey data characterizing feasibility, acceptability, frequency and length of calls. The process and narrative data were qualitatively summarized. The descriptive results of the outcome data are presented for the two study groups (statistical comparisons were not made). For analysis, self-reported adherence and MPR were dichotomized as ≥95% and <95%, respectively, with ≥95% considered acceptable.

## RESULTS

Between January and September 2017, 60 pregnant women living with HIV were screened, and 40 were found eligible and consented to participate. Participants were randomized evenly to the intervention and control arms (*n* = 20 each) (Fig. 1).

### Baseline characteristics

The mean age of the participants was 24.85 (SD = 3.59) years (Table 2). All participants were married, and only 15% had completed secondary school education. Groups were comparable in socio-demographic (education, marital status, religion) and HIV-related variables. Overall, 72% of the participants had a second- or higher-order pregnancy, 80% had been tested for HIV as part of the antenatal testing policy of the PMTCT program, 67% had a spouse living with HIV and 95% had disclosed their status to their spouse.

**Table 2:** Socio-demographic characteristics

	**Total**	**Intervention**	**Control**
Age—mean years (SD)	24.85 (3.59)	24.9 (3.93)	24.8 (3.33)
Education			
Never attended school	30 (12/40)	30 (6/20)	30 (6/20)
1–5 classes of school	17.5 (7/40)	25 (5/20)	10 (2/20)
6–11 classes of school	37.5 (15/40)	35 (7/20)	40 (8/20)
Completed 12 classes of school	15 (6/40)	10 (2/20)	20 (4/20)
Marital status			
Currently married	100 (40/40)	100 (20/20)	100 (20/20)
Religion			
Hindu	87.5 (35/40)	80 (16/20)	95 (19/20)
Muslim	10 (4/40)	15 (3/20)	5 (1/20)
Other (specify)	2.5 (1/40)	5 (1/20)	0 (0/20)
Gestational age at baseline—mean weeks (SD)	28.87 (2.96)	29.6 (3.1)	28.15 (2.7)
ART naïve or experienced			
ART naïve: no ART/no NVP	50 (20/40)	45 (9/20)	55 (11/20)
PMTCT experienced: recd NVP for previous pregnancy	22.5 (9/40)	25 (5/20)	20 (4/20)
ART experienced: taking ART for HIV treatment	27.5 (11/40)	30 (6/20)	25 (5/20)
Number of months on ART—mean months (SD)	11.95 (23.34)	18.05 (30.29)	5.85 (11.09)
Pregnancy history			
First pregnancy	27.5 (11/40)	25 (5/20)	30 (6/20)
Second- or higher-order pregnancy	72.5 (29/40)	75 (15/20)	70 (14/20)
Reason for taking HIV test			
Was feeling sick repeatedly	5 (2/40)	5 (1/20)	5 (1/20)
Husband/partner tested HIV positive	12.5 (5/40)	15 (3/20)	10 (2/20)
Pregnancy—PPTCT	80 (32/40)	80 (16/20)	80 (16/20)
Other	2.5 (1/40)	0 (0/20)	5 (1/20)
Partner’s HIV status			
HIV positive	67.5 (27/40)	65 (13/20)	70 (14/20)
HIV negative	30 (12/40)	30 (6/20)	30 (6/20)
Don’t know	2.5 (1/40)	5 (1/20)	0 (0/20)
Disclosed HIV status to partner/spouse			
Yes	95 (38/40)	100 (20/20)	90 (18/20)
No	5 (2/40)	0 (0/20)	10 (2/20)

### Feasibility and fidelity

Sixty-seven percent (*n* = 40) of the screened women (*n* = 60) were enrolled. Overall retention was 82% (33/40) with higher retention in the intervention arm (90%) versus the control (75%) (Fig. 1, Table 3).

**Table 3:** Feasibility and fidelity of the intervention content and delivery

**Indicators**	**Intervention**	**Control**
The ratio of eligible study participants to those enrolled	67% (*n* = 40 of 60 participants who were screened) enrolled into the study (across treatment and attention control arms)—20 randomized to each arm	
Number of scheduled study visits completed	***n* (%)**	***n* (%)**
Baseline	20 (100)	20 (100)
36–40 weeks pregnancy	14 (70)	15 (75)
Post-delivery	20 (100)	18 (90)
6 weeks postpartum	19 (95)	17 (85)
12 weeks postpartum	18 (90)	15 (75)
Retention between baseline and endline	***n* (%)**	***n* (%)**
(12 weeks postpartum)	18 (90)	15 (25)
Reason for premature drop-out	***n* (%)**	***n* (%)**
Death	1 (5)	0
Loss to Follow Up	1 (5)	1 (5)
Withdrawal from study	0	1 (5)
Unable to conduct endline	0	3 (15)
Calls made on schedule & level of participation		
Calls in the ANC period:		
Calls completed as planned	***n* (%)**	***n* (%)**
Calls initiated by participants	7 (78%)	5 (70%)
	0	0
Mean (SD) of call length (in minutes)	**x- (SD)**	**x- (SD)**
Calls in the PNC period:	19.27 (13.05)	6.67 (3.12)
Calls completed as planned	***n* (%)**	***n* (%)**
Calls initiated by participants	6 (95%)	6 (80%)
	1	0
Mean of call length (in minutes) (SD)	**x- (SD)**	**x- (SD)**
	13.61 (5.8)	5.66 (2.0)
Topics/content discussed on calls/(%)	**Topics discussed in the ANC & PNC periods**	**Topics discussed in the ANC period**
	Adherence (e.g. reminder strategies) (92%)	General wellbeing (99%)
	Moods/Depressive symptoms (91%)	Blood pressure (80%)
	Stigma/Disclosure (87%)	Urine test (80%)
	Symptom/Side effect management (86%)	Ultrasound during pregnancy (80%)
	HIV transmission (41%)	Iron and folic acid supplements (80%)
	Appointments (77%)	Plans for baby’s birth at home or hospital (80%)
	Social Support (73%)	Plans for breastfeeding (80%)
	Infant care (54%)	Other topics (20%)
	Other topics (4%)	
		**Topics discussed in the PNC period:**
		General wellbeing (100%)
		Where the baby was delivered (100%)
		Type of delivery (100%)
		Contraception for family planning (100%)
		How the baby is doing (94%)
		Feeding (94%)
		Child vaccination at birth (BCG, OPV and Hep B) (94%)
		Next vaccination due (94%)
		Other topics (10%)
Adverse events	***n* (%)**	
	1 (5%)—unrelated to the intervention, appropriate authorities informed	0

Participants received scheduled mobile phone counselling calls throughout the antenatal and postpartum periods. In the antenatal phase, both groups received an average of five calls. Participants in the intervention arm attended 76% of the scheduled antenatal calls, compared to 74% in the control arm. In the postpartum period, the intervention group received more calls on average (seven calls) than the control group (five calls). Attendance rates for postpartum calls were also higher in the intervention arm (91%) compared to the control arm (77%) (Table 3).

Call duration differed notably between groups. During the antenatal period, calls averaged 19.27 minutes (SD = 13.5) in the intervention group and 6.67 minutes (SD = 3.12) in the control group. In the postpartum period, intervention calls averaged 13.61 minutes (SD = 5.8), while the control calls averaged 5.66 minutes (SD = 2.0).

In the intervention arm, counsellors demonstrated a high level of fidelity to the intervention protocol, with strong alignment between prescribed topics and those proposed in the intervention protocol and those discussed with participants (Table 3). Key areas of focus included adherence (92%), mood and depressive symptoms (91%), stigma and disclosure issues (87%), management of ART-related symptoms (86%) and social support (73%).

In the control arm, counsellors successfully adhered to the non-HIV counselling protocol. Calls focused on antenatal care topics prior to delivery and postpartum health education. Eighty percent of the calls focused on blood pressure checks, urine tests, ultrasound tests, iron and folic acid supplementation, plans to breastfeed, delivery and infant-related issues in the postpartum period [experiences related to place of delivery and type of delivery (100%), contraception advice (100%), infant health (100%), infant feeding with a focus on breastfeeding or bottle milk (94%), and infant immunization (94%) (Table 3)].

### Acceptability

The mobile phone counselling was well received by the participants (Table 3). A majority of participants reported that it ‘helped them a lot’—76% in the intervention group and 69% in the control group (Table 4). Additionally, 65% of the intervention participants reported feeling more confident about caring for themselves while living with HIV, compared with 50% of the control group participants.

**Table 4:** Acceptability of the intervention

**Question**	**Intervention (*N* = 17)**	**Control (*N* = 15)**
	**% (*n*)**	**% (*n*)**
How helpful/useful were the calls to you overall?		
A little	0 (0)	6% (1)
Some	24% (4)	25% (4)
A lot	76% (13)	69% (11)
How much did the calls help you to feel more confident about caring for yourself while living with this illness?		
A little	0% (0)	6% (1)
Some	35% (6)	44% (7)
A lot	65% (11)	50% (8)
Were there any topics that were not discussed on the calls or only discussed little that you would have liked to discuss more?		
No	82% (14)	87% (14)
Did you like having a phone as part of being in this study?		
Yes	94% (16)	100% (16)
Did the having the phone or receiving the calls create problems for you in any way?		
Yes	35% (6)	19% (3)
Was the quality of the phone connection ok most of the time		
Yes	65% (11)	56% (9)
Did you like that the counsellor called you at scheduled times?		
Yes	100% (17)	100% (16)
Would you have preferred if you could have made all of the calls to the counsellor?		
No	70% (12)	69% (11)
Do you think the calls lasted long enough?		
Yes	94% (16)	100% (16)
Do you think the calls were frequent enough?		
Yes	100% (17)	100% (16)
Do you think the calls would be helpful to other women living with your illness?		
Yes	100% (17)	100% (15)
Do you think some women would prefer to have calls from someone other than a counsellor?		
Yes	59% (10)	62% (10)
No	41% (7)	37% (6)
Do you think some women would like to talk to other women living with the illness or learn about the experiences of other women living with your illness by phone?		
Yes	94% (16)	87% (14)
Is there anything that you might suggest that would help to improve or make the calls better/more useful to women like you?		
No	76% (13)	69% (11)

Use of the mobile phones was also positively regarded, with 94% of the intervention group and 100% of the control group expressing satisfaction. Despite this, 35% of the intervention participants and 19% of the control participants reported that receiving a phone created some difficulties. Nevertheless, all participants in both arms (100%) appreciated having the counsellor call at scheduled times (Table 4).

Most participants preferred not to initiate the calls themselves (70% intervention, 69% control). Phone connectivity challenges were noted by a substantial portion of the participants (65%, intervention, 56%, control).

Participants across both groups found that the frequency and duration of the calls were appropriate, and all (100%) thought the calls would benefit other women living with HIV. Many also thought that women would might value opportunities to connect other women living with HIV or learn more about the experiences of other women living with HIV—94% in the intervention group and 87% in the control group.

### Psychosocial and biologic outcomes

#### Adherence

Mean self-reported adherence over the 4 days prior to clinic visits remained high (≥95%) in both arms throughout the study period (Table 5). However, improvement in ‘TIs’—a lapse in ART for >7 days—were more pronounced in the intervention group. At baseline, 25% of participants in the intervention group reported TIs compared to 10% in the control arm. By endline, these rates had decreased to 6% and 7%, respectively. Additionally, pharmacy refill data indicated stronger results in the intervention group. The MPR at endline was 88% for the intervention group, compared to 62% in the control group.

**Table 5:** Psychosocial and biologic indicators at baseline and follow-up.

	**Baseline**		**Follow Up**							
			**36–40-week pregnancy**		**Within 1 week of delivery**		**6 weeks postpartum**		**12 weeks postpartum (Endline)**	
	**Intervention (*n* = 20)**	**Control (*n* = 15)**	**Intervention (*n* = 14)**	**Control (*n* = 15)**	**Intervention (*n* = 20)**	**Control (*n* = 18)**	**Intervention (*n* = 19)**	**Control (*n* = 17)**	**Intervention (*n* = 18)**	**Control (*n* = 15)**
EPDS (mean, SD)	18.35 (3.49)	18.15 (2.56)	11.85 (6.19)	13.66 (5.53)	7.65 (6.32)	5.61 (3.95)	8.73 (7.95)	9.35 (7.57)	6.33 (6.74)	6.73 (5.24)
BIPQ score (mean, SD)	49.1 (3.33)	47.85 (7.31)					44.52 (5.75)	45.52 (5.4)	43.38 (6.74)	42.53 (4.42)
Internal stigma (mean, SD)	46 (4.73)	43.05 (3.66)					44.31 (4.55)	44.58 (4.24)	45.11 (3.95)	46.73 (3.8)
Adherence (mean, SD)	100 (0)	94.73 (13.38)	100 (0)	98.33 (6.45)			93.42 (23.33)	95.58 (18.19)	95.83 (12.86)	100 (0)
Treatment interruption^a^	25%	10%	0%	7%			26.3%	11.8%	6%	7%
CD4 count (mean, SD)	439.47 (181.83)	430.45 (220.35)					488.78 (284.98)	545.17 (252.3)		
Viral load (% >200)	20%	30%					16%	7%		
Mean MPR^b^									88%	62%

a % who had interruptions between assessments.

b Medication possession ratio.

#### Biologic indicators

A corresponding positive trend in biological outcomes was observed in both arms. At baseline, elevated HIV-1 RNA (>200 copies/ml) were found in 25% of the intervention group and 30% of the control arm. By 6 weeks postpartum, these rates had declined to 16% in the intervention group and 7% in the control group. Similarly, CD4 counts increased over the study period in both groups, reflecting improvements in immune status consistent with effective ART (Tables 5 and 6).

**Table 6:** Biologic indicators at baseline and endline across groups

**Indicator**	**Total % (*n*/*N*)**		**Intervention group % (*n*/*N*)**		**Control group % (*n*/*N*)**	
	**Baseline**	**Endline**	**Baseline**	**Endline**	**Baseline**	**Endline**
Viral load						
<20 copies/ml	60 (24/40)	79 (27/34)	60 (12/20)	74 (14/19)	60 (12/20)	87 (13/15)
20–200 copies/ml	15 (6/40)	9 (3/34)	20 (4/20)	11 (2/19)	10 (2/20)	7 (1/15)
201–1000 copies/ml	10 (4/40)	6 (2/34)	5 (1/20)	5 (1/19)	15 (3/20)	7 (1/15)
>1000 copies/ml	15 (6/40)	6 (2/34)	15 (3/20)	11 (2/19)	15 (3/20)	0 (0/15)
CD4 count						
≥500 cells/mm^3	33 (13/39)	56 (20/36)	37 (7/19)	53 (10/19)	30 (6/20)	59 (10/17)
≥500 cells/mm^3	66 (26/39)	44 (16/36)	63 (12/19)	47 (9/19)	70 (14/20)	41 (7/17)

#### Depression, stigma and illness perception

Mean depression scores, as measured by the EPDS, declined substantially and similarly in both groups from baseline to endline. In the intervention arm, scores decreased from 18.35 (SD = 3.49) to 6.33 (SD = 6.74), while in the control arm, scores decreased from 18.15 (SD = 2.56) to 6.70 (SD = 5.24) (Table 5). Depression scores dropped sharply between enrollment and the first week postpartum, rose slightly at 6 weeks postpartum and then declined again by endline.

Perceptions of illness, measured using the Brief Illness Perception Questionnaire (B-IPQ), also improved in both groups. In the intervention group, scores declined from 49.1 at baseline to 43.3 at the endline; in the control group, scores declined from 47.8 to 42.5 (Table 5), indicating reduced illness-related concern.

Stigma scores followed different trajectories across groups. At baseline, the intervention group reported higher perceived stigma (mean = 46) compared to the control group (mean = 43). However, by endline, stigma had increased in the control group (46.7) (Table 5).

## DISCUSSION

This pilot study demonstrated the feasibility and acceptability of delivering mobile phone counselling to perinatal women living with HIV and depression in India. The intervention was successfully integrated into routine perinatal care and ART services, achieving strong participant retention and high client satisfaction. Mobile phone counselling was shown to be a promising, low-cost and sustainable strategy to reach and engage women in mental health and HIV care programs, particularly in resource-constrained settings.

Beyond demonstrating feasibility, the study identified actionable approaches to support improvements in women’s health behaviours and retention across the continuum of care. These findings are especially relevant as national programs seek to implement and sustain World Health Organization (WHO) recommendations for combination ART from pregnancy through breastfeeding and lifelong ART continuation [46]. Overall, the results suggest that mobile phone counselling may offer a highly practical, scalable approach to enhance continuity of care and improve health outcomes among women with HIV and comorbid depression.

The intervention was feasible to implement and well received. Retention at 12 weeks postpartum was higher in the intervention group, as was participation in scheduled calls. Fidelity to the intervention was also high, with strong alignment between call content and the theory-guided counselling framework. Participants in the intervention group reported greater satisfaction, with over three-quarters stating that the intervention helped them ‘a lot’ and nearly two-thirds expressing increased confidence in managing their self-care.

The mobile phone counselling intervention was grounded in understanding participants’ individual illness perceptions and tailoring support accordingly. The counsellor identified areas of strength and risk and provided health information and support contextualized to the patient’s illness perceptions, situational context and priorities. Importantly, women in both arms expressed interest in connecting with other women living with HIV. Given that many women living with HIV report feelings of isolation [47], this suggests potential for leveraging mobile phone counselling platforms to explore peer-based or group support formats in the future.

Although this study was not powered to assess efficacy, exploratory findings revealed positive trends that align with the study’s conceptual framework. Depressive symptoms were common at baseline with two-thirds of those screened meeting the threshold for elevated depressive symptoms using the EPDS—a pattern consistent with findings from previous studies in similar settings [48, 49]. Mean depression scores declined in both the intervention and control arms to less than half of baseline levels by study end. While the study was not powered to assess efficacy, it was interesting to observe that depression improved in both arms. While the reduction in symptoms across both groups limits conclusions about the intervention’s distinct effect, the overall improvement is notable.

The attention-control design, which included provision of a mobile phone and scheduled mobile phone calls, may have minimized between-group differences. It may also indicate that consistent engagement, regular follow-up and the opportunity to express their feelings may have contributed to improved mental health regardless of the specific content of the calls. Although counsellors in the control group adhered to the non-HIV-related content protocol, even general discussions around pregnancy and newborn care may have offered emotional relief and support that benefited participants’ mental health.

Additional exploratory outcomes included ART adherence, illness perceptions and stigma. Both study arms reported high levels of self-reported adherence throughout the study. However, the intervention group demonstrated greater reductions in treatment interruptions and achieved higher MPR scores at endline, suggesting improved consistency in medication access and use. These trends are encouraging, particularly in light of the well-established association between adherence and viral suppression [17].

Illness perceptions improved in both groups, indicating reduced illness-related concern. However, stigma outcomes revealed a trend toward divergence between groups: there was a signal toward improvement in the intervention group; stigma scores increased in the control group. Given the persistent role of stigma as a barrier to care for women living with HIV, this finding is promising. The patient-centred, theory-informed counselling approach delivered in the intervention targeted internalized stigma by addressing key psychosocial domains, such as disclosure, coping strategies and self-efficacy.

Taken together, these findings highlight the potential value of structured, individualized support—even in settings where formal efficacy testing is not the primary goal. They underscore the importance of integrating psychosocial and emotional support into HIV care, particularly for women navigating both perinatal challenges and chronic illness. Future studies powered to assess efficacy will be essential to confirm and expand upon these promising trends.

An important consideration for scalability is the affordability and sustainability of the intervention. By leveraging widely available low-cost mobile phone technology and existing health workforce capacity, BEST-ma-CARE minimizes infrastructure and capital investment requirements. The use of non-specialist counsellors, combined with remote delivery, reduces personnel and facility costs while maintaining personalized care. These attributes position the intervention as a cost-effective and adaptable model for health systems in low-resource settings, particularly where mental health services are limited.

### Limitations of the study

This study had certain limitations. First, as a pilot feasibility and acceptability trial, it was not powered to assess efficacy. Second, the analyses were limited to participants who remained in the study; if attrition was non-random, this may have introduced bias. Third, several of the outcomes were measured with self-report, which may be subject to social desirability bias. Nevertheless, the use of both subjective and objective measures (e.g. pharmacy refill data, viral load testing) adds strength to the overall assessment.

## CONCLUSION

The findings from this study underscore the need for contextually appropriate strategies to prevent, identify and manage depression in perinatal women living with HIV in LMICs. Accordingly, we advocate for policies that integrate mental health support—such as brief depression screening and individualized counselling—into routine HIV care. Our findings also point to the potential value of expanding mental health interventions through peer or group-based models, particularly when delivered through mobile phone platforms. The one-to-one, mobile phone counselling intervention, BEST-ma-CARE, was found to be both feasible and acceptable. This approach offers a scalable and adaptable model that could be integrated into clinical services to enhance patient engagement, improve mental health outcomes and support HIV treatment adherence. Future research should evaluate the cost and effectiveness of BEST-ma-CARE in a fully powered trial and explore opportunities for its adaptation and implementation across diverse healthcare settings.

## Data Availability

The data that support the findings of this study were collected in India and are subject to local data protection and sovereignty regulations. In compliance with Indian law and institutional policy, individual-level research data must remain stored within India. De-identified data may be made available to qualified researchers upon reasonable request, pending approval by the relevant Indian ethics committees, the Health Ministry’s Screening Committee (HMSC), and the collaborating Indian institution. Data access requests will be reviewed to ensure alignment with the study’s informed consent procedures, Indian regulatory requirements and NIH data sharing policies. Requests should be directed to the corresponding author and will be considered on a case-by-case basis by the study’s principal investigator and relevant institutional review board to ensure compliance with ethical and legal standards.
